# Supplementary material for: Cholesterol esters form supercooled lipid droplets whose nucleation is facilitated by triacylglycerols
Source: Nat Commun. 2023 Feb 17;14:915. doi: 10.1038/s41467-023-36375-6 (PMC9938224; doi:10.1038/s41467-023-36375-6)
Supplement: Supplementary file 1 — Supplementary Information File [file 41467_2023_36375_MOESM1_ESM.pdf]

Supplementary Information

Cholesterol esters form supercooled lipid droplets whose nucleation is facilitated by triacylglycerols

Calvin Dumesnil[1*], Lauri Vanharanta[2,3*], Xavier Prasanna[4*], Mohyeddine Omrane[1], Maxime Carpentier[1], Apoorva Bhapkar[1], Giray Enkavi[4], Veijo T. Salo[2,3,5], Ilpo Vattulainen[4#], Elina Ikonen[2,3#], Abdou Rachid Thiam[1#]

[1]Laboratoire de Physique de l'École normale supérieure, ENS, Université PSL, CNRS, Sorbonne Université, Université Paris Cité, F-75005 Paris, France

[2]Department of Anatomy and Stem Cells and Metabolism Research Program, Faculty of Medicine, University of Helsinki, Helsinki, Finland

[3]Minerva Foundation Institute for Medical Research, Helsinki, Finland

[4]Department of Physics, University of Helsinki, Helsinki, Finland

[5]Structural and Computational Biology Unit, European Molecular Biology Laboratory (EMBL), Heidelberg, Germany

[*]Equal contribution

[#] Corresponding author (thiam@ens.fr, elina.ikonen@helsinki.fi, ilpo.vattulainen@helsinki.fi)

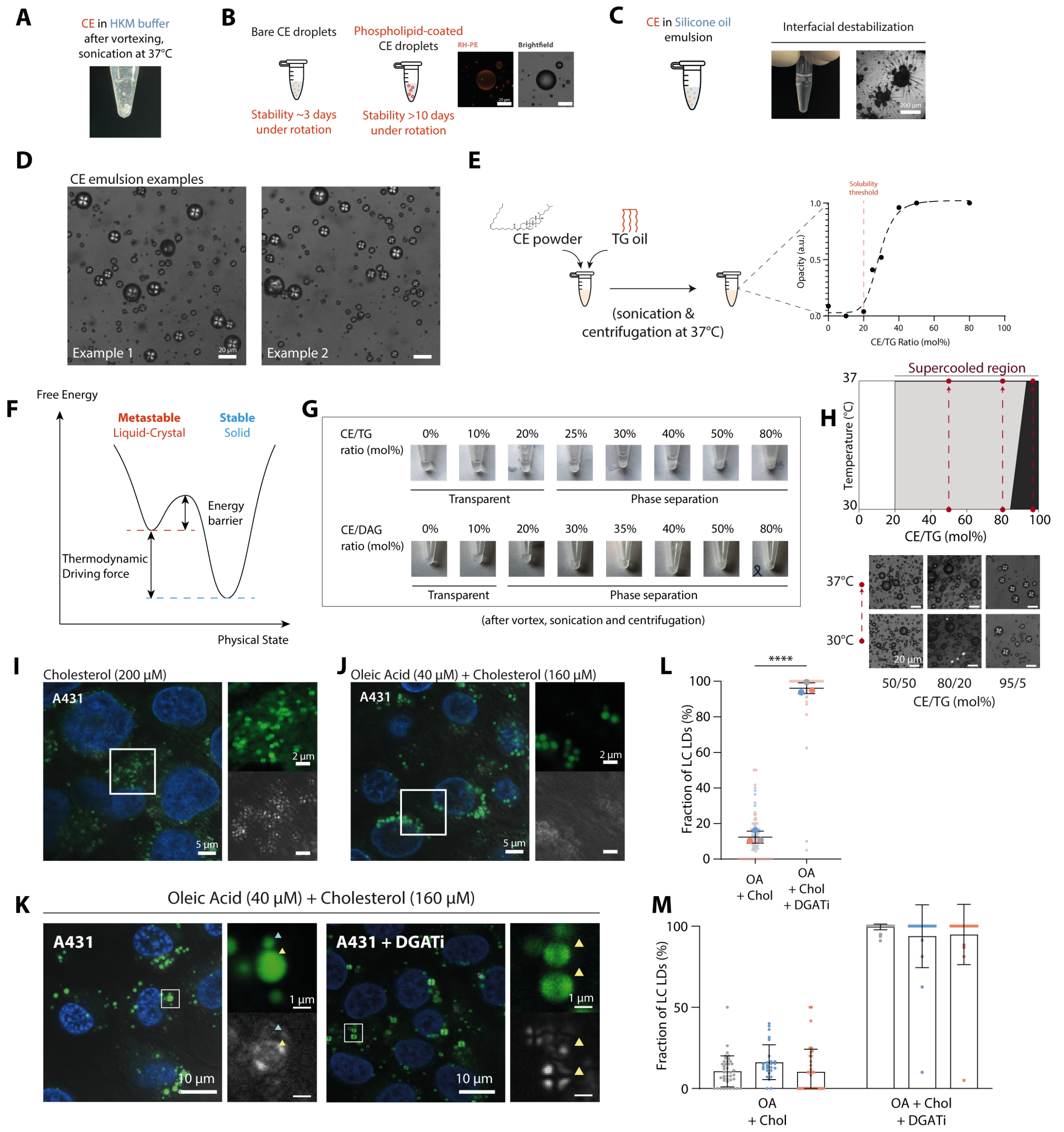

Supplementary Figure 1. Characterization of CE LDs in vitro and in cells.

(A) Picture of CE and HKM buffer after mixing, vortexing, sonicating and centrifugation at 37°C. CE did not get emulsified through the process.

(B) Schematic representations of the stability of pure CE emulsions under rotation. The emulsions were stable for ~3 days for bare droplets, and more than 10 days for phospholipids-coated CE emulsion. Microscope image of the phospholipid-coated emulsion.

(C) Microscope image of a CE in Silicone oil emulsion. Droplets exhibit interfacial destabilization.

(D) Two examples of pure CE emulsion droplets showing liquid crystalline organization in the majority.

(E) Schematic representation of the CE and TG mixing protocol. CE and TG were mixed, sonicated and centrifugated at 37°C. **Right,** quantification of the solubility of CE in TG by measuring the opacity of the solution.

(F) Schematic representation of Liquid-Crystal to Solid phase transition. The Liquid crystalline phase is metastable as it is not the absolute minimum of the Free Energy. The difference in Free Energy between the two phases is the driving force of the phase transition. However, an energy barrier has to be overcome for the phase transition to happen.

(G) Pictures of CE & TG and CE & DAG mixtures. The mixtures were transparent below 20 mol% of CE in TG and below 10% in DAG. Phase separation occurred from 25% in TG and 10% in DAG.

(H) Examples of polarized light images of CE/TG emulsions (50/50, 80/20, 95/5) heated from 30°C to 37°C.

(I) Example image of A431 cells imaged after 24h of Cholesterol (200 µM) feeding. Bodipy was added upon imaging for LD labelling.

(J) Example image of A431 cells imaged after 24h of Oleic Acid (40 µM) + Cholesterol (160 µM) feeding. Bodipy was added upon imaging for LD labelling.

(K) A431 cells imaged after 24h of Oleic Acid (40 µM) + Cholesterol (160 µM) (Left) or Oleic Acid (40 µM) + Cholesterol (160 µM) and DGAT inhibitors feeding (Right). Bodipy was added upon imaging for LD labelling and further analysis (I).

(L) Analysis of (H). Fraction of liquid crystalline LDs, Mean+/- SD. N=41, 26 and 37 cells for 160 µM Cholesterol + 40 µM Oleic Acid. N=54, 25, and 27 cells for 160 µM Cholesterol + 40 µM Oleic Acid + DGATi. Each color point represents a data point from a replicate. The experiment was independently repeated three times with similar results. **** $p<0.0001$ two-tailed Nested t tests.

(M) Separate view of the distributions of the 3 replicates merged in (L). Each colour represents a replicate.

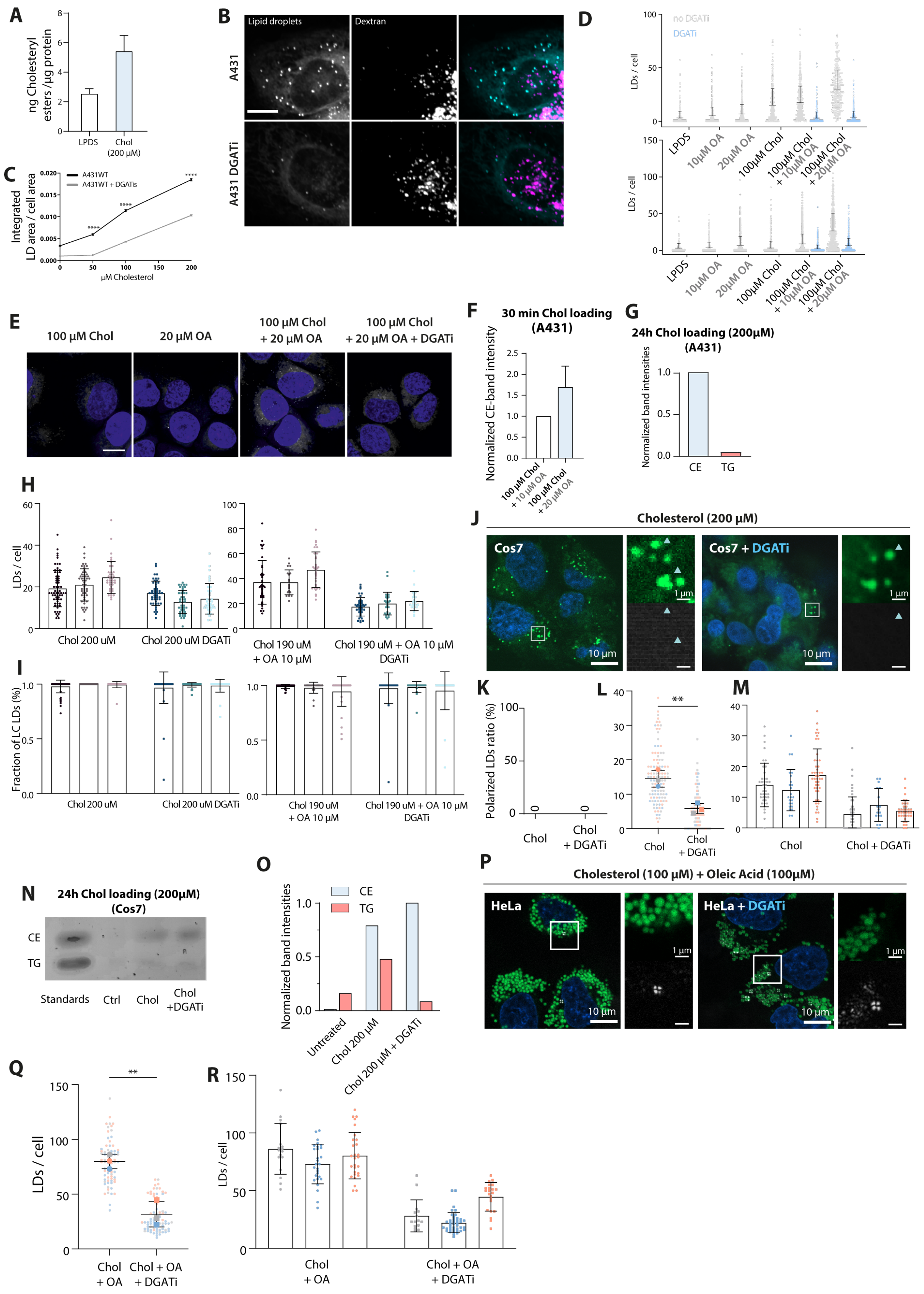

Supplementary Figure 2. Impact of TG on CE LD formation in cells.

(A) Biochemical analysis of CE levels by thin layer chromatography (TLC). Lipids were extracted from A431WT cells starved in 5% LPDS for 4 days + DGATi for overnight and

loaded with 200 µM cholesterol for 1 h; Mean of 5 measurements from 2 experiments +/- SD.

(B) Lipid droplet dye LD540 is not prominently staining lysosomes. Maximum intensity projections of confocal images of A431WT cells starved in 5% LPDS for 4 d + DGATi for overnight and loaded with 100 µM cholesterol cyclodextrin for 1 h +/- DGATi. Cells were supplemented with 40 µg/mL Alexa Fluor 647-Dextran (Thermo Fisher Scientific, D22914) for overnight and during cholesterol loading to label the endo-lysosomal compartment. Cells were fixed and stained with LD540 before imaging. Scale bar = 10 µm.

(C) Quantification of the LD integrated area for the different cholesterol concentrations studied in Figure 2A. n= 899 cells for 0 µM, 944 cells for 0 µM + DGATi, 734 cells for 50 µM, 1016 cells for 50 µM + DGATi, 547 cells for 100 µM, 948 cells for 100 µM + DGATi, 705 cells for 200 µM, and 864 cells for 200 µM + DGATi condition. Mean+/- SEM. **** $p < 0.0001$, two-tailed student's t-test.

(D) Quantification of lipid droplet numbers from two individual replicate experiments for the experiment presented in Figure 2C. Top: n= 495 cells for LPDS, 546 cells for 10 µM OA, 498 cells for 20 µM OA, 302 cells for 100 µM chol, 388 cells for 100 µM chol + 10 µM OA, 312 cells for 100 µM chol + 20 µM OA, 569 cells for 100 µM chol + 10 µM OA + DGATi, and 547 cells for 100 µM chol + 20 µM OA + DGATi condition. Mean+ SD and all individual data points. Bottom: n= 137 cells for LPDS, 353 cells for 10 µM OA, 677 cells for 20 µM OA, 649 cells for 100 µM chol, 1083 cells for 100 µM chol + 10 µM OA, 617 cells for 100 µM chol + 20 µM OA, 482 cells for 100 µM chol + 10 µM OA + DGATi, and 323 cells for 100 µM chol + 20 µM OA + DGATi condition. Mean+ SD and all individual data points.

(E) Representative maximum intensity projection images of the cholesterol/oleic acid conditions quantified in Figure 2C. Scale bar = 10 µm. The experiment was independently repeated two times with similar results.

(F) Biochemical analysis of CEs by TLC from A431WT cells starved in 5% LPDS for 4 d + DGATi for overnight and loaded with cholesterol and oleic acid + DGATi as indicated for 30 min. Mean of 6 measurements from 3 experiments +/- SD, 1 corresponds to 3.3 ng CE/µg protein.

(G) Representative result of TLC analysis of CE and TG from 24h cholesterol-fed A431. Two experiments were done.

(H) Separate view of the distributions of the 3 replicates merged in Figure 2G. Each color represents a replicate.

(I) Separate view of the distributions of the 3 replicates merged in Figure 2F. Each color represents a replicate.

(J) Representative observation of Cos7 cells imaged after 24h of cholesterol (200 µM) (Left) or cholesterol (200 µM) with DGATs' inhibitors presence (Right). Bodipy was added upon imaging for LD labelling and further analysis (C) and (D). The experiment was independently repeated three times with similar results.

(K) Analysis of (I) of the aggregated experiments. Fraction of liquid crystalline LDs.

(L) Analysis of (I) of the aggregated experiments. Number of LDs per cell. N=41, 24 and 46 cells for 200 µM Cholesterol. N=54, 17, and 35 cells for 200µM Cholesterol + DGATi.

Each color point represents a data point from a replicate. The experiment was independently repeated three times with similar results. ** p=0.0055 two-tailed Nested t test.

(M) Separate view of the distributions of the 3 replicates merged in (K). Each color represents a replicate.

(N) Representative TLC analysis of CE and TG of Cos7 cells fed for 24h with cholesterol or cholesterol in the presence of DGATi. Two independent experiments were done.

(O) Quantification of experiment shown in J.

(P) Representative image of HeLa cells imaged after 24h of cholesterol (100 µM) + oleic acid (100 µM) (Left) or cholesterol (100 µM) + oleic acid (100 µM) in the presence of DGAT inhibitors (Right). Bodipy was added upon imaging for LD labelling. The experiment was repeated more than four times.

(Q) Analysis of the number of LDs in cells in the presence or absence of DGATi, when loaded with oleic acid and cholesterol (200 µM each). N=17, 28 and 28 cells for 200 µM Cholesterol + 200µM Oleic Acid. N=15, 40, and 22 cells for 200µM Cholesterol + 200µM Oleic Acid + DGATi. Each color point represents a data point from a replicate. The experiment was independently repeated three times with similar results. ** p=0.0035 two-tailed Nested t test. Separate view of the distributions of the 3 replicates merged in (P). Each color represents a replicate.

(R) Separate view of the distributions of the 3 replicates merged in (P). Each color represents a replicate.

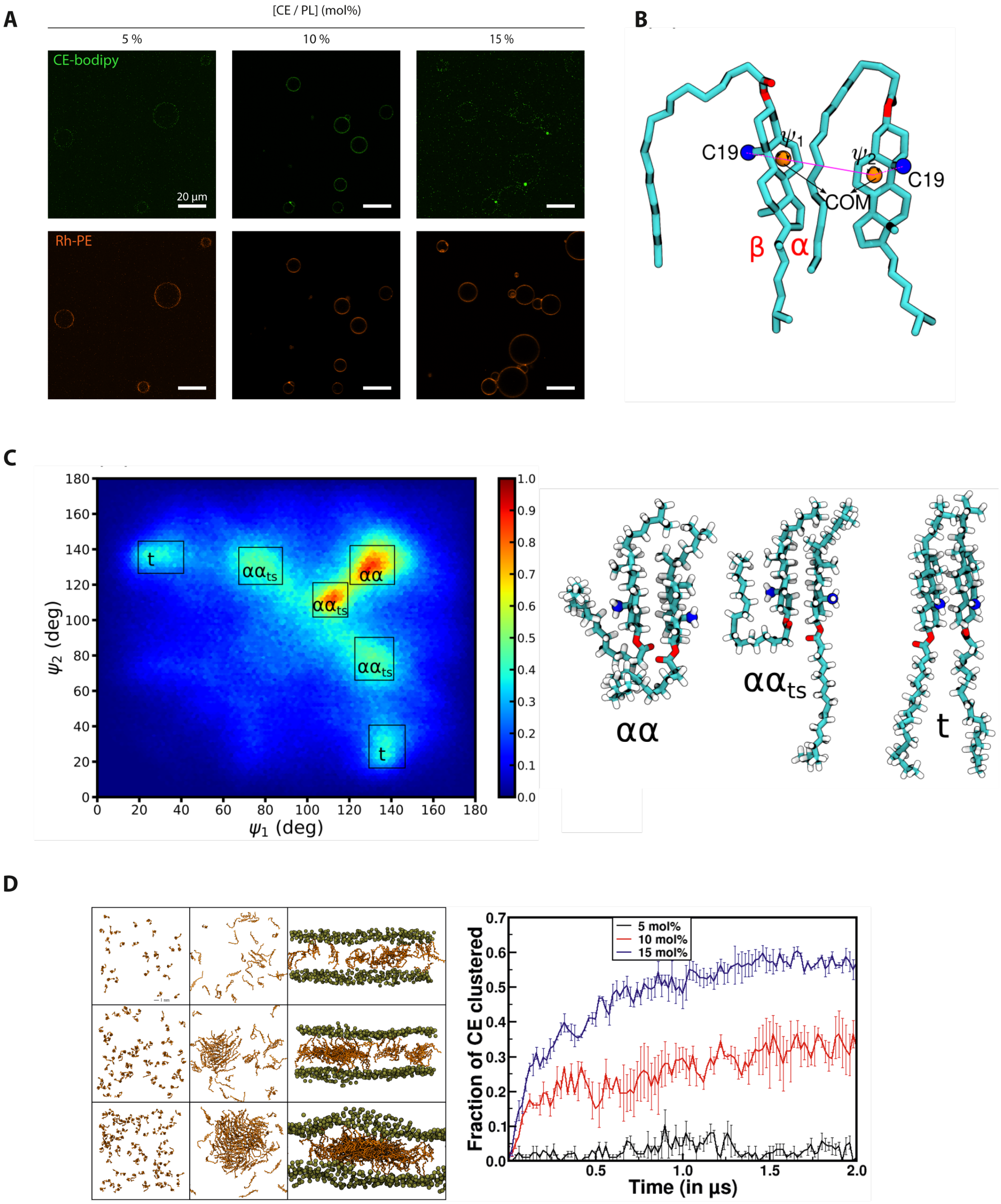

Supplementary Figure 3. CE behavior in membranes.

(A) Image of Rh-PE labeled PC/PE (70/30) GUVs containing 5, 10 and 15 mol% of CE-Bodipy labelled CE. GUVs were deposited on a BSA-coated coverslip.

(B) Definition of generalized Crick angles (Ψ1, Ψ2) as order parameters characterizing the structures of CE packing. C19 atoms (blue) identify the beta-face of CE and COM denotes the center of mass of CE ring B.

(C) A quantitative plot showing the relative population of different CE packing conformations in the cluster.

(D) Left: Top-view distribution of CE (orange) in DOPC/DOPE (70/30) bilayers at 5 mol% (top row), 10 mol% (middle row) and 15 mol% CE (bottom row) during the beginning of the simulation (left column) and at the end (2 microseconds) of the simulation (middle column). A side-view of the systems at the end of the simulation period is also shown (right column). Right: Fraction of CE clustered in a DOPC/DOPE (70/30) bilayer. The values have been obtained from a single simulation for each system.

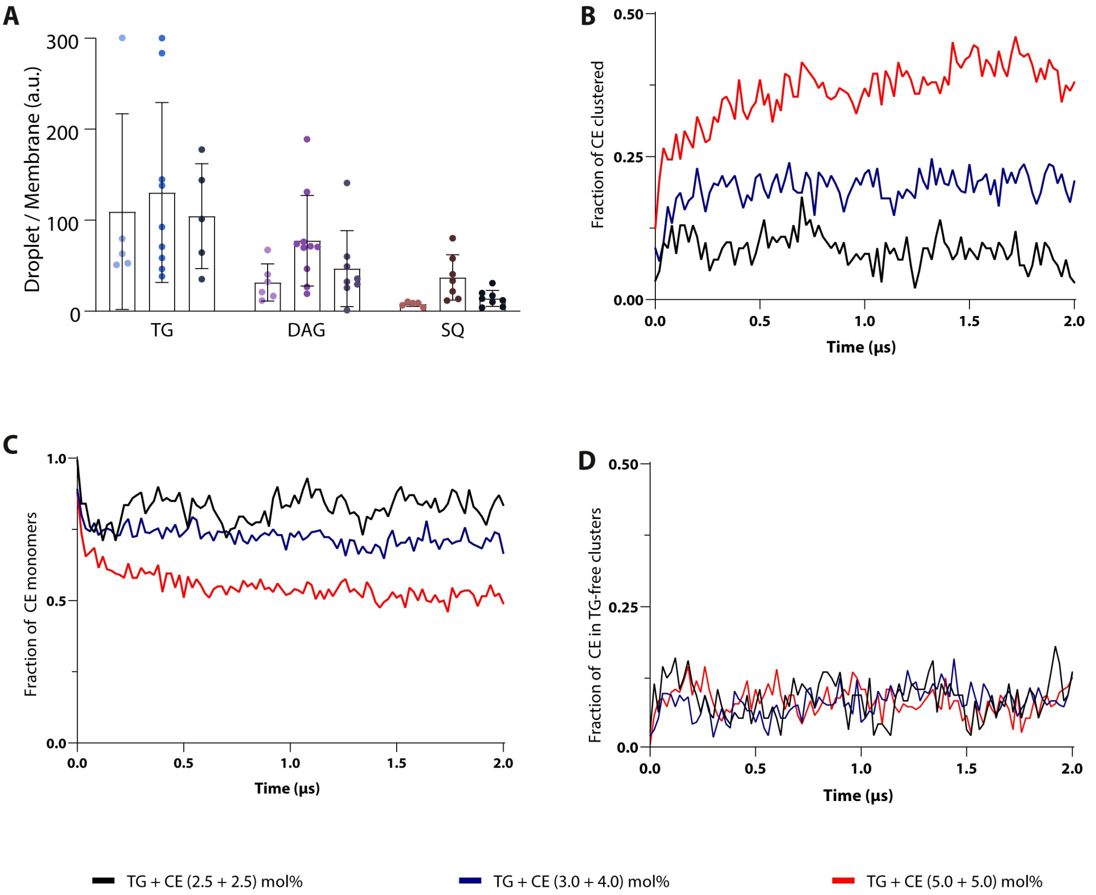

Supplementary Figure 4. Partitioning of CEs into TGs.

(A) Separate view of the distributions of the 3 replicates merged in Figure 4B. Each color represents a replicate.

(B) Plot showing the fraction of CE molecules clustered in the bilayers (with 2.5 mol% each of TG and CE, 3 mol% TG and 4 mol% CE, ER, and 5.0 mol% each of TG and CE) during the simulations. The data have been averaged over three simulation repeats.

(C) Plot showing the fraction of free CE monomers in the bilayer during the simulations. The data have been averaged over three simulation repeats.

(D) Plot showing the fraction of CE molecules in TG-free clusters in the bilayer during the simulations. The data have been averaged over three simulation repeats.

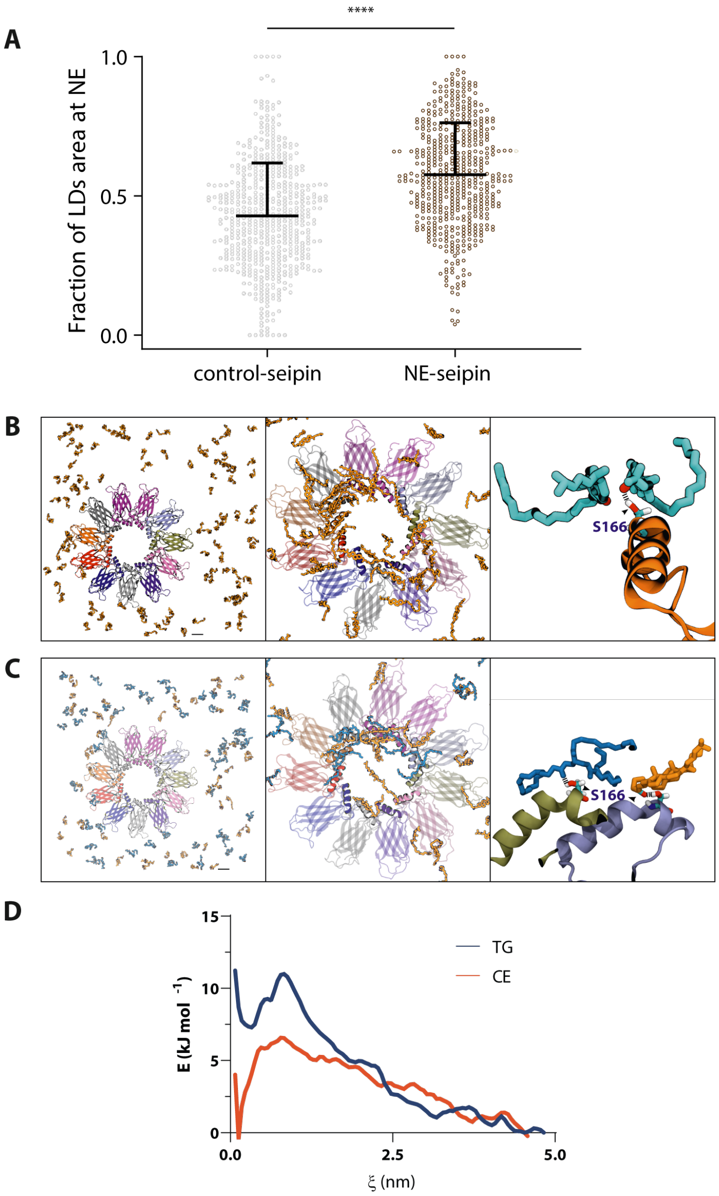

Supplementary Figure 5. Impact of seipin and TG on CE LD formation.

(A) Lipid droplet area overlapping with nuclei quantified as fraction of LDs area at NE. An individual experiment similar to the experiment presented in Figure 5B, except cholesterol loading for 1 h. Control n= 570 and NE-trap n=526 cells. Mean+ SD and all individual data points. **** p< 0.0001, two-tailed student's t-test.

(B) Top-view representation of CE molecules (5 mol%, shown in orange) around the seipin oligomer (luminal domain only) associated with a model ER membrane. **Left:** At the beginning (t = 0). **Middle:** At the end (t = 5 µs) of the simulation period (zoomed in to show CE association with α2-α3 helices). Each protomer is shown with a different color. **Right:** A more close-up view showing the dimerization of a CE molecule not bound to seipin with another CE molecule interaction with residue S166 on a seipin protomer. Black bars represent the interacting atoms between CE molecule and residue S166.

(C) Top-view representation of CE (2.5 mol%, shown in orange) and TG (2.5 mol%, shown in blue) molecules around the seipin oligomer (luminal domain only) associated with a model ER membrane, **Left:** At the beginning (t = 0). **Middle:** at the end (t = 5 µs) of the simulation period (zoomed in to show CE association with α2-α3 helices). Each protomer is shown with a different color. **Right:** A more close-up view showing the interaction of a single CE and a single TG molecule with the residue S166 on two different seipin protomers. Black bars represent the interacting atoms between the residues and neutral lipids.

(D) Free energy profiles for TG and CE with respect to their binding in the immediate vicinity of S165 and S166. The free energy barrier for binding/unbinding is about 4 kBT for both TG and CE. Attention should be paid to the free energy barrier needed to overcome to unbind a complex.
