# Supplementary material for: Cholesterol esters form supercooled lipid droplets whose nucleation is facilitated by triacylglycerols
Source: Nat Commun. 2023 Feb 17;14:915. doi: 10.1038/s41467-023-36375-6 (PMC9938224; doi:10.1038/s41467-023-36375-6)
Supplement: Supplementary file 3 — Description for Additional Supplementary Files [file 41467_2023_36375_MOESM3_ESM.pdf]

Description of Additional Supplementary Files

Supplementary Movie 1
TG droplets explode and wet the water-air interface with no particular feature. The movie displays three times faster.

Supplementary Movie 2
CE droplets explode and wet the water-air interface, displaying onion rings reminiscent of liquid instabilities. The movie displays three times faster.

Supplementary Movie 3
Visualization of a CE droplet at water-air interface crystalizing upon contacting a nucleation seed.
