# Supplementary material for: Cholesterol esters form supercooled lipid droplets whose nucleation is facilitated by triacylglycerols
Source: Nat Commun. 2023 Feb 17;14:915. doi: 10.1038/s41467-023-36375-6 (PMC9938224; doi:10.1038/s41467-023-36375-6)
Supplement: Supplementary file 7 — Reporting Summary [file 41467_2023_36375_MOESM7_ESM.pdf]

nature portfolio

Corresponding author(s): Abdou Rachid THIAM
Elina IKONEN
Ilpo Vattulainen

Last updated by author(s): Jan 5, 2023

Reporting Summary

Nature Portfolio wishes to improve the reproducibility of the work that we publish. This form provides structure for consistency and transparency in reporting. For further information on Nature Portfolio policies, see our Editorial Policies and the Editorial Policy Checklist.

Statistics

For all statistical analyses, confirm that the following items are present in the figure legend, table legend, main text, or Methods section.

n/a	Confirmed	
[ ]	[x]	The exact sample size (*n*) for each experimental group/condition, given as a discrete number and unit of measurement
[ ]	[x]	A statement on whether measurements were taken from distinct samples or whether the same sample was measured repeatedly
[ ]	[x]	The statistical test(s) used AND whether they are one- or two-sided<br>*Only common tests should be described solely by name; describe more complex techniques in the Methods section.*
[ ]	[x]	A description of all covariates tested
[ ]	[x]	A description of any assumptions or corrections, such as tests of normality and adjustment for multiple comparisons
[ ]	[x]	A full description of the statistical parameters including central tendency (e.g. means) or other basic estimates (e.g. regression coefficient) AND variation (e.g. standard deviation) or associated estimates of uncertainty (e.g. confidence intervals)
[ ]	[x]	For null hypothesis testing, the test statistic (e.g. *F*, *t*, *r*) with confidence intervals, effect sizes, degrees of freedom and *P* value noted<br>*Give P values as exact values whenever suitable.*
[x]	[ ]	For Bayesian analysis, information on the choice of priors and Markov chain Monte Carlo settings
[x]	[ ]	For hierarchical and complex designs, identification of the appropriate level for tests and full reporting of outcomes
[x]	[ ]	Estimates of effect sizes (e.g. Cohen's *d*, Pearson's *r*), indicating how they were calculated

Our web collection on statistics for biologists contains articles on many of the points above.

Software and code

Policy information about availability of computer code

Data collection	Zen Software (2.3) was used for data collection.
Data analysis	FiJi (Fiji is just ImageJ) for image analysis, PRISM 7 for data visualization, Python-based Custom codes for microscope imaging processing

For manuscripts utilizing custom algorithms or software that are central to the research but not yet described in published literature, software must be made available to editors and reviewers. We strongly encourage code deposition in a community repository (e.g. GitHub). See the Nature Portfolio guidelines for submitting code & software for further information.

Data

Policy information about availability of data

All manuscripts must include a data availability statement. This statement should provide the following information, where applicable:

- Accession codes, unique identifiers, or web links for publicly available datasets
- A description of any restrictions on data availability
- For clinical datasets or third party data, please ensure that the statement adheres to our policy

Data supporting the study are available within the manuscript, supplements, or from the authors upon request.

Human research participants

Policy information about studies involving human research participants and Sex and Gender in Research.

Reporting on sex and gender	n/a
Population characteristics	n/a
Recruitment	n/a
Ethics oversight	n/a

Note that full information on the approval of the study protocol must also be provided in the manuscript.

Field-specific reporting

Please select the one below that is the best fit for your research. If you are not sure, read the appropriate sections before making your selection.

☒ Life sciences ☐ Behavioural & social sciences ☐ Ecological, evolutionary & environmental sciences

For a reference copy of the document with all sections, see nature.com/documents/nr-reporting-summary-flat.pdf

Life sciences study design

All studies must disclose on these points even when the disclosure is negative.

Sample size	The sample sizes were not predetermined. Sample sizes were determined sufficient by ruling out the null hypothesis using statistical tests (unpaired t-tests).
Data exclusions	No data was excluded from the analyses
Replication	All attempts at replication were successful, quantitative experiments were done in triplicates to ensure the reproducibility. Should not it be possible, very large sample size was used.
Randomization	Randomization was not applicable to the study since it is based on cells. Instead, control experiments were made with equivalent cell populations for each replicate.
Blinding	Blinding was not applicable to our study since data were analyzed either using unbiased softwares or manually analyzed by the experimenter.

Reporting for specific materials, systems and methods

We require information from authors about some types of materials, experimental systems and methods used in many studies. Here, indicate whether each material, system or method listed is relevant to your study. If you are not sure if a list item applies to your research, read the appropriate section before selecting a response.

Materials & experimental systems

n/a	Involved in the study	
☒	☐	Antibodies
☐	☒	Eukaryotic cell lines
☒	☐	Palaeontology and archaeology
☒	☐	Animals and other organisms
☒	☐	Clinical data
☒	☐	Dual use research of concern

Methods

n/a	Involved in the study	
☒	☐	ChIP-seq
☒	☐	Flow cytometry
☒	☐	MRI-based neuroimaging

Eukaryotic cell lines

Policy information about cell lines and Sex and Gender in Research

Cell line source(s)	Human epithelial carcinoma cells (A431) (BioWhittaker, Lonza, Verviers, Belgium), HeLa (human cervical carcinoma obtained from the American Type Culture Collection), Cos7 cells obtained from Dr. Marie-Françoise Bourgeade (Inserm UMR-S 1193) from the American Type Culture Collection.
Authentication	None of the cell lines used were authenticated.
Mycoplasma contamination	All cell lines tested negative for Mycoplasma contamination.
Commonly misidentified lines (See ICLAC register)	No commonly misidentified cell lines were used in this study.
